# A Preliminary Study on the Cultural Competence of Nurse Practitioners and Its Affecting Factors

**DOI:** 10.3390/healthcare10040678

**Published:** 2022-04-03

**Authors:** Tsui-Ting Liu, Miao-Yen Chen, Yu-Mei Chang, Mei-Hsiang Lin

**Affiliations:** 1 Department of Nursing, Taipei Veterans General Hospital, National Taipei University of Nursing and Health Science, Taipei 112303, Taiwan; liucatting@gmail.com; 2 School of Nursing, National Taipei University of Nursing and Health Sciences, Taipei 112303, Taiwan; miaoyen@ntunhs.edu.tw; 3 Department of Nursing, Asia Eastern University of Science and Technology, New Taipei City 22061, Taiwan; fk001@mail.aeust.edu.tw

**Keywords:** cultural competence, nurse practitioners, nursing education

## Abstract

Cultural competence refers to a healthcare provider’s ability to consider cultural factors that affect an individual’s health and attitudes toward disease and disability. Nurse practitioners (NPs) are increasingly important in healthcare, practicing culturally competent care strategies to improve the quality of patient care. The aim of this study was to explore cultural competence and its related factors among NPs. A cross sectional study design with a structured questionnaire survey was used. Purposive sampling was employed, for which 86 NPs were recruited from a medical center in northern Taiwan. A *T*-test, one-way ANOVA, and Pearson’s product-moment correlation coefficient were used for data analysis. The results were as follows: (1) overall, the total score for cultural competence was above-average, with a score of 3.75; (2) years of experience as a NP was found to have a statistically significant correlation with overall clinical competence, with *r* = 0.23, *p* < 0.05; (3) there were significant differences in clinical awareness and cultural sensitivity related to the clinical ladder system (*t* = −2.42, *p* = 0.01; *t* = −2.04, *p* = 0.04). The findings of this study can provide information for directors of medical institutions to design an in-service educational program for NPs to enhance their cultural competence and nursing quality.

## 1. Introduction

Nursing philosophy is the foundation of nurse practitioner (NP) training [1]. NPs have come into existence because of their demand in the health care system, and their role and areas of practice vary according to the health care needs and legal and medical systems in different countries or districts [2,3]. NPs provide medical care equivalent to that of physicians and facilitate access to healthcare [4]. Currently, in Taiwan, there are approximately 7000 NPs, the majority of whom practice in various acute care settings [3,4,5]. The NP scope of practice includes the two following major parts: direct patient care and indirect patient care. The top three direct patient care activities include performing a physical assessment, discussing the treatment plan with physicians, and taking patient history. The top three indirect patient care activities are charting, coordinating patient care, and assisting with paperwork related to medical interventions [5].

Taiwan has been driving the internationalization of medical services since 1995. However, many foreign nationals have experienced problems and disparities in healthcare services when seeking medical treatments [5]. These disparities arise from multifarious causes, which include the issue of language barriers, but more often concern the problem of cultural differences [6]. Leininger [7] suggested that professional health care providers should adapt to the changes in social structure and improve their cultural congruence to provide healthcare services that can cater to the patients’ cultural needs. Cross et al. [8] define cultural competence as “a set of congruent behaviors, attitudes, and policies that come together in a system, agency, or amongst professionals and enable that system, agency or those professionals to work effectively in cross-cultural situations”. Cultural competence in nursing focuses largely on the ability to compare and contrast the healthcare beliefs, values, and cultural lifestyles of different individuals, and to provide them with culturally appropriate and beneficial care [9]. There are many advantages in the provision of culturally appropriate care. For example, it empowers patients and makes them feel respected and more compliant with treatment plans, in turn improving their prognosis [10]. Many studies highlighted that NPs provide medical care equivalent to that of physicians and facilitate access to healthcare [11,12,13]. Igrarshi et al. [4], interviewed NPs in Japan and discovered that NPs can improve the quality of healthcare by proposing practical solutions to problems faced by patients and medical organizations. Nursing practitioners in Taiwan were developed in the context of a shortage in medical practitioners resulting from the change in the medical context after the establishment of National Health Insurance in 1995 [5]. Their primary responsibility is to cooperate with medical practitioners and offer complete and continuous medical and nursing care to patients. Apart from possessing considerable knowledge and experience about a specific group of diseases to provide patients of that group with higher quality nursing services, they are also in charge of education, consultation, research, policy amendments, and administrative management [14,15,16]. During the provision of healthcare services, if NPs can take into consideration the differences in languages, health perception, lifestyles, and cultural diversity among patients and respect each patient’s native cultural beliefs and behavior, they can not only improve their healthcare quality and the patients’ satisfaction, but also eliminate the existing inequality in healthcare services [17]. 

Cultural competence is not an innate characteristic, and instead requires learning [12,15,16]. Campinha-Bacote [18] proposed in a study that cultural competence is composed of five aspects, including cultural awareness, cultural knowledge, cultural skills, cultural encounter, and cultural desire. This definition has been acknowledged by various empirical studies, especially in the field of nursing research [19,20]. Liang et al. [21] conducted an investigation into the multicultural competence of nursing staff in Taiwan and categorized cultural competence into four aspects, which include cultural awareness, cultural knowledge, cultural sensitivity, and cultural skills. The research results showed that the nursing staff’s cultural knowledge and skills were yet to be improved. Cultural awareness refers to the ability to realize in a meaningful manner that one’s own cultural viewpoints are different from those of others and to scrutinize one’s own professional and cultural values, in order to know more about any personal understandings and bias towards foreign cultures. When we grasp an understanding of how our fixed cultural values and beliefs affect our interactions with patients, our cultural sensitivity becomes sharpened. On the other hand, cultural knowledge refers to the ability to seek and obtain knowledge related to different cultural groups, including their health beliefs, health-seeking behavior, perception about illness and morality, and the treatments that they deem effective. Cultural sensitivity is the ability to sympathize with, trust, respect, and accept the beliefs and values of different cases, and to give weight to the patient’s culture and its influence on their health-seeking behavior. Cultural skills stand for the ability to leverage tools and resources to develop communication skills, gather information about the subject’s cultural background, and conduct a cultural assessment accordingly for the purpose of fulfilling the needs arising from the patient’s background [15,21].

Clinical practice must rely on multicultural practice and learning to help NPs reinforce their critical thinking and understand the variances among cases and medical teams in an intricate cultural context. Thus, they can learn to respect cultural differences and broaden their cultural horizon, in turn fulfilling the expectations of each case and medical team [16,21]. In Taiwan, the differences in dialects, living regions, religious beliefs, and cultural backgrounds influence the health patterns and perception of individuals [21]. Building a nurse-patient relationship with effective communication and trust is the key to improving healthcare quality. Therefore, the ability to accept patients’ cultures and overcome language barriers should be prioritized urgently. When NPs conduct health assessments during their first encounter with patients, any failure in considering their cultural diversity, lifestyles, and health perception because of language differences, miscommunication, or cultural insensitivity will hinder their provision of culturally appropriate healthcare services [15,16].

Culture is the motive behind individuals’ behavior during the process of playing roles in ever-developing social backgrounds [18]. It has a rather high level of specificity. A reflection on the content of in-service education currently provided in clinical medical institutions reveals a lack of culture-related topics [22]. Nevertheless, it is clinically common to encounter patients that are new habitants or old-aged people accompanied by foreign caretakers, who request to carry with them a lucky charm obtained from a Chinese temple before surgeries, or whose family members demand to perform religious rituals in the ward as required by the condition of the patient. Countless healthcare behaviors of ethnic or cultural diversity can be found in the medical context. The research suggests that in order to provide culturally competent care, NPs should consider every possible non-medical problem that can occur in each case, such as how their background knowledge related to dietary or living styles could affect the patient’s health [23]. Furthermore, due to the high degree of specificity and individuality of cultures, NPs cannot design nursing programs according to uniform standards when interacting with patients from different cultures. Hence, our research aimed to investigate the question, “What is NP’s capacity for multicultural care in clinical practice in Taiwan?” To answer this question, this study aims at exploring the status quo of NPs’ cultural competence and its affecting factors to obtain domestic data from Taiwan. The results provide a reference for the directors of medical institutions to design in-service education schemes for NPs that can enhance their cultural competence and the quality of their healthcare services.

## 2. Materials and Methods

### 2.1. Design and Sample

This study employed a cross-sectional design. A purposive sample is one in which characteristics are defined for a purpose that is relevant to the study [24]. This study purposely examines the cultural competence and its related factors among NPs, because we do not know what competencies NPs perform or how effective they are in their work in clinical practice in Taiwan. Thus, purposive sampling was utilized to collect data. A total of 86 NPs who had completed the nurse practitioners training program, and had at least one-year’s practical experience as a clinical NP were recruited from a medical center located in the north of Taiwan. The sample size was estimated using the G*power statistical software package version 3.1.2 (http://www.gpower.hhu.de/, accessed on 13 March 2022). A sample size of at least 82 has 80% power to detect a medium effect size of 0.3. An alpha level of 0.05 was used. A total of 90 questionnaires were issued. After sample collection and blank and invalid questionnaires were removed (the same answer for all tests), 86 valid samples were recovered for data analysis. The valid response rate was 95.55%.

### 2.2. Measurements

#### 2.2.1. Demographic Data Questionnaire

The demographic characteristics questionnaire included gender, age, marital status, education level, seniority, clinical ladder system, years of experience as an NP, service division, attended courses related to cultural diversity and experience of studying abroad [21].

#### 2.2.2. Nurses’ Multicultural Caring Competence Scale (NMCCS)

The NMCCS was used to assess NPs’ cultural competence. A total of 29 items were grouped into the following four domains: cultural awareness, cultural knowledge, cultural sensitivity, and cultural skills. The NMCCS was devised by Liang et al. [21]. It divides cultural competence into four aspects, with a total of 29 questions (seven on cultural awareness, eight on cultural knowledge, three on cultural sensitivity, and 11 on cultural skills). Cronbach’s alpha for the overall scale and the four subscales are between 0.91 and 0.97, showing the comprehensive psychometric properties [21]. In addition, the authors of the original scale constructed the scale based on Taiwanese nursing staff, who had a similar culture to the participants in this study, so this study used NMCCS. The four domains were scored on a 5-point Likert scale, from 1 being “strongly disagree” to 5 being “strongly agree”. The higher the score, the higher the level of cultural competence in the corresponding aspect based on the participant’s self-assessment [21]. The original scale was designed to measure the cultural competence of general nursing staff, but as this study targets nurse practitioners, some options on basic attributes have been amended, and their content validity was tested by five nursing educators, nursing experts, and experienced NPs. The content validity index (CVI) was 0.98. On the other hand, the overall Cronbach’s α of this scale was 0.93.

### 2.3. Data Analysis

SPSS for Windows 20.0 software (SPSS, Inc., Chicago, IL, USA) was used for data analysis. The frequency, percentage, mean, SD, *t*-test, one-way ANOVA, Pearson product correlation, and multiple regression were utilized. The *p* value for the significance level was less than 0.05.

### 2.4. Ethical Considerations

The study was approved by the institutional review board of Taipei Veterans General Hospital in Taiwan (Approval Code: 2017-02-003BC). The participants were informed that the research process would not involve any risk or comorbidity. The study was conducted after obtaining signed informed consent from each participant. Completed questionnaires were placed into a questionnaire return box in each department to be retrieved by the research assistant.

## 3. Results

### 3.1. Demographic Characteristics of the Participants

The study sample contained 86 participants, and 94.2%of them were females. The mean age of all subjects was 43.48 years (SD = 5.54). The NPs’ mean seniority and years of experience as an NP were 20.62 years (SD = 5.92), and 8.62 years (SD = 5.99), respectively. Fifty-seven participants (66.3%) were married. The highest educational certification held by the majority of participants was a bachelor’s degree, accounting for 70.9%. Sixty participants had a clinical ladder system that was above N3 (69.8%). In terms of service division, 37 participants (43%) were in surgery wards. A large proportion of the subjects (*n* = 82, 95.3%) had not attended courses related to cultural diversity. Seventy-five participants (65.2%) had exercise habits. In terms of studying abroad, eight of them had experienced studying abroad (9.3%) (Table 1).

### 3.2. The Distribution of the Cultural Competence

The NPs’ cultural competence was measured by the Nurses’ Multicultural Caring Competence Scale (NMCCS) in this study. Four dimensions were scored. The mean score of the overall cultural competence was 3.75 (SD = 0.41). According to the results, the mean score in cultural awareness was 4.41 (SD = 0.42), with the highest score in “People from different cultural backgrounds tend to differ in their values”. In terms of cultural sensitivity, the mean score was 4.32 (SD = 0.45); the highest scoring statement was “I respect the differences between the different ethnic cultures”. As for cultural skills, the mean score was 3.42 (SD = 0.57); the statement with the highest score was “I can explain the possible cultural relevance of the client’s beliefs or behavior of the health/disease”. Lastly, in terms of cultural knowledge, the mean was 3.42 (SD = 0.62), with the highest score in “I can explain the possible correlation between a patient’s health/illness beliefs or behavior and his culture”. (Table 2).

### 3.3. Association between Participants’ Demographics and Variables and Cultural Competence Score

Pearson’s product correlation was computed to evaluate the relationship among age, seniority, years of experience as an NP and overall cultural competence. The results showed that years of experience as an NP was significantly statistically correlated with overall cultural competence with *r* = 0.22, *p* < 0.05 (Table 3).

## 4. Discussion

The nurse practitioners sampled in this study scored an average of 3.75 (SD = 0.41) in their overall cultural competence, which was an above-average performance. Among the four related aspects, cultural awareness received the highest score, while cultural skills had the lowest. This result is consistent with the finding of Dobrowolska et al. [25] and Liang et al. [21], which reported that nurses had acquired a certain level of cultural awareness and sensitivity, but their cultural knowledge and skills were yet to be improved. The finding of this study shows that the NPs’ seniority correlated significantly and positively with their cultural skills, and their cultural competence was similar to the study by Juan, Zhuang, Yong, and Rong [26], which reported that years of work experience would directly affect the nurses’ self-efficacy for cultural competence. Significant differences were found between the NPs’ positions in the clinical ladder system and their cultural awareness and sensitivity. This finding is consistent with that of a previous study [27].

The results of this study showed a lack of statistical significance between cultural competence and the variables of gender, marital status, education level, attended courses related to cultural diversity, experience of study abroad and one’s service division. This finding is contradictory with a number of previous studies [10,21,28,29]. A possible explanation for this is the high homogeneity among the nurse practitioners in this study. Their cultural competence was unaffected by their differences in personal background attributes, learning, and work experiences. Such results could be regarded as a positive phenomenon. Many academic papers have proposed several factors that affect the cultural competence of nurses, including participation in multicultural nursing, ethnic minority background, exchange study, frequency of interacting with culturally different people in the workplace or daily life, education level, experience of caring for multiple ethnic groups [10,28,30], cultural diversity training [29], identical religious beliefs with patients [29], language skills [28,31], cross-cultural communication skills [32,33], clinical experience in foreign countries [34], and ethnocentrism [33]. Additionally, according to Purnell [35], the major influences that shape peoples’ worldview and the degree to which they identify with and adhere to their cultural group of origin are called the primary and secondary characteristics of culture. Among them, marital status is one of the secondary characteristics. A somewhat different finding emerged in the present study. The result of this study showed that there was not a significant difference between cultural competence and marital status. Nevertheless, the finding was contradictory with a study by Khodaveisi et al. [36], which reported significant differences between cultural competence and marital status. Possible explanations for this finding are the differences between the questionnaires used in the studies and the populations it studied. Furthermore, the years of work experience, professional titles, income, ethnicity, and employment status will also exert a direct impact on the nurses’ self-efficacy for cultural competence [26]. In addition, despite the fact that only 4.7% of the NPs in this study had participated in a multicultural curriculum, they had high self-assessment scores in cultural awareness and sensitivity, and an above-average overall cultural competence. This showed that the NPs’ cultural competence might be attributed to quotidian life and work experience. Unfortunately, data about previous work experience was not collected in this study. In comparison, the NPs’ self-assessment scores in cultural knowledge and skills were relatively low. This perhaps indicated their deficiency in educational training that can remind them how to acquire more cultural knowledge and skills. Therefore, to tackle this existing issue, consideration should be given to promoting in-service education.

In recent years, many nursing universities have begun to pay more attention to developing students’ cultural competence, and hence, have started establishing courses related to cross-cultural nursing in the university curriculum [28,37]. As illustrated from the previous study, the NPs who were more culturally competent had received cultural competence training, had the experience of performing nursing work in different cities, and could communicate in the patients’ language were able to derive higher patient satisfaction [22]. Another study also showed that NPs’ participation in conferences that emphasized cultural awareness had positive effects on their cultural knowledge and competence. Attending workshops that build cultural competence with cultural skills is also beneficial to enhancing nurse–patient communication and cooperation. Improving NPs’ cultural competence can improve their communication with patients and lead to better health outcomes for them [27]. As the research targets of this study were NPs that joined the profession at an earlier time, most of them did not participate in a multicultural curriculum when they were receiving standard nursing education. After entering the workforce, their in-service education was also short of training that could aid them in improving their cultural competence. In addition, several studies have reported on the importance of mutual education among NPs and other medical professionals such as general physicians and other health care professionals [38,39]. Therefore, this study urges education and training units in hospitals and related societies to face the fact that multicultural ethnic groups have been increasing in number. More learning opportunities and course options should be provided for NPs to improve their cultural competence and consequently provide patients with a more culturally appropriate assessment, nursing service and care.

Chinese has been designated as the official language of Taiwan, in which Minnan and Hakka are two major dialects. As English is not our official language, it is unlikely that everyone can speak fluently with an English-speaking foreign national. All of the participants in this study had received post-secondary education or above, half of whom had obtained a verification for English language proficiency, had studied Western medicine, and wrote medical records in English at work. In fact, they had all acquired a certain degree of English proficiency. However, some academic papers pointed out that nursing education was not the main source for developing cultural competence [40]. The emphasis of nursing education regarding cultural knowledge and skills should be placed on developing the ability to explore and seek knowledge actively, instead of teaching concrete knowledge and skills about cultural diversity [28]. As a result, when NPs encounter non-English-speaking patients from foreign countries, what they need to know is how to search for reliable resources and appliances to assist them in understanding the patient’s needs. The development of multicultural knowledge is a continuous process [18]. If institutions can take advantage of in-service education and incorporate more multicultural courses to help NPs gain cultural knowledge, NPs can definitively harness their cultural skills more efficiently and consequently produce a more culturally appropriate healthcare service.

This study had several limitations. Firstly, this study was restricted to one medical center in northern Taiwan and did not use a large sample. Consequently, the results may not be generalizable to NPs employed in other hospitals. Additionally, purposive sampling can cause low validity and reliability. Based on the results of this study, a larger sample size is recommended to strengthen the significance of the findings. Moreover, generalizations from a purposive sample to a wider population is possible only if the sample was randomly drawn from that population, something which is suggested for further study.

## 5. Conclusions

This study investigated cultural competence among NPs. With reference to the results of this study, the overall cultural competence of NPs was above-average, with the highest score in cultural awareness, as well as significant differences between their clinical ladder and their cultural awareness and skills. It is advised that related studies in the future should extend the research area to other districts or regional hospitals to investigate whether NPs’ cultural competence is related to the regions to which they belong, urban–rural disparities, or the nature of the hospitals for which they work. Finally, apart from an increase in the number of participants, the researchers also suggest that future studies adopt a qualitative research design and specify the affecting factors of NPs’ cultural competence and their multicultural learning experience, in order to facilitate a further assessment of their needs for in-service education about cross-cultural nursing.

## Figures and Tables

**Table 1:** Demographic characteristics of the participants.

Variables	*n*	%	Mean (SD)
Gender			
Female	81	(94.2)	
Male	5	(5.8)	
Age			43.48 (5.54)
Marital status			
Unmarried	29	(33.7)	
Married	57	(66.3)	
Education level			
Bachelor’s degree	61	(70.9)	
Master’s degree	25	(29.1)	
Clinical Ladder System			
≥N3	60	(69.8)	
≤N2	26	(30.2)	
seniority			20.62 (5.92)
Years of experience as a NPs			8.62 (5.99)
Service division			
Internal medicine	26	(30.2)	
Surgery	37	(43)	
Obstetrics & Gynecology	9	(10.5)	
Intensive care unit	14	(16.3)	
Attended courses related to cultural diversity			
no	82	(95.3)	
Yes	4	(4.7)	
Experience of studying abroad			
Yes	8	(9.3)	
no	78	(90.7)	

**Table 2:** The distribution of cultural competence among participants.

Items	M	SD
Cultural awareness	4.41	0.42
A person’s beliefs or behaviors are influenced by its cultural background	4.47	0.50
People from different cultural backgrounds tend to differ in their values	4.50	0.56
Most people’s health/disease beliefs or behaviors are influenced by cultural background	4.48	0.52
Cultural knowledge	3.42	0.62
I can tell the specific health problems between different ethnic groups	3.55	0.83
I can collect knowledge and information about health/disease of different cultures	3.44	0.71
I can explain the possible cultural relevance of the client’s beliefs or behavior of the health/disease	3.57	0.71
I can compare the health/disease beliefs of different cultural background clients	3.47	0.74
I can understand the care needs of different cultural background cases	3.45	0.80
Cultural sensitivity	4.32	0.45
I respect the differences between the different ethnic cultures	4.43	0.56
I am keen to advatages in the health care methods of my clients and appreciate them	4.36	0.52
Cultural skill	3.42	0.57
I can use communication skills in clients of different cultural backgrounds	3.67	0.67
I can understand the nonverbal expressions of different cultural background cases	3.53	0.79
When performing nursing activities, I can meet the needs of different cultural background clients	3.52	0.66
I can explain the impact of culture on client’s health/disease beliefs or behavior	3.45	0.77
Before performing the nursing activities, I will collect the cultural background information related to the clients	3.52	0.66
Overall cultural competence	3.75	0.41

**Table 3:** Descriptive Statistics and associations between cultural competence and participants’ demographics.

Variables	M	SD	1.	2.	3.	4.	5.	6.	7.	8.
1. Age	43.48	5.54	1							
2. seniority	20.62	5.92	0.89 **	1						
3. Years of experience as a NPs	8.62	5.99	0.44 **	0.50 **	1					
4. Cultural awareness	4.41	0.42	0.09	0.09	0.02	1				
5. Cultural knowledge	3.42	0.62	0.08	0.10	0.19	0.14	1			
6. Cultural sensitivity	4.32	0.45	−0.03	−0.01	0.12	0.52 **	0.23 *	1		
7. Cultural skills	3.42	0.57	0.08	0.14	0.24 *	0.06	0.78 **	0.14	1	
8. Overall Cultural competence	3.75	0.41	0.10	0.14	0.23 *	0.40 **	0.89 **	0.41 **	0.89 **	1

* *p* < 0.05; ** *p* < 0.01. The results showed that there were significant differences in clinical awareness and cultural sensitivity related to the clinical ladder system, with *t* = −2.43, *p* = 0.01; −2.03, *t* = −2.43, *p* < 0.04, respectively (Table 4).

**Table 4:** Differences among participants’ demographic variables and cultural competence score.

Variables	Cultural Awareness		Cultural Knowledge		Cultural Sensitivity		Cultural Skills		Overall Cultural Competence	
	Mean	±SD	Mean	±SD	Mean	±SD	Mean	±SD	Mean	±SD
Gender ^a^										
Male	4.37	0.37	3.42	0.33	4.60	0.43	3.45	0.38	3.78	0.25
Female	4.42	0.42	3.44	0.63	4.31	0.45	3.42	0.59	3.75	0.42
*t*	−0.25		−0.00		1.36		0.11		0.15	
*p* value	0.79		0.99		0.17		0.90		0.88	
Marital status ^a^										
Unmarried	4.43	0.44	3.35	0.72	4.41	0.43	3.40	0.64	3.73	0.49
Married	4.43	0.41	3.46	0.56	4.28	0.46	3.43	0.54	3.77	0.36
*t*	−0.53		−0.73		1.22		−0.26		−0.40	
*p* value	0.59		0.46		0.22		0.78		0.69	
Education level ^a^										
Bachelor’s degree	4.39	0.39	3.38	0.52	4.27	0.43	3.38	0.54	3.72	0.34
Master’s degree	4.46	0.48	3.54	0.81	4.45	0.49	3.52	0.65	3.85	0.53
*t* (*p*) value	−0.64		−0.90		−1.61		−1.01		−1.35	
*p* value	0.52		0.37		0.10		0.31		0.18	
Clinical Ladder System ^a^										
≥N3	4.48	0.44	3.40	0.63	4.39	0.46	3.37	0.60	3.75	0.42
≤N2	4.26	0.34	3.48	0.60	4.17	0.41	3.53	0.50	3.76	0.38
*t* (*p*) value	−2.43		0.52		−2.03		1.16		0.72	
*p* value	0.01		0.60		0.04		0.24		0.94	
Attended courses related to cultural diversity ^a^										
No	4.41	0.42	3.41	0.60	4.32	0.45	3.42	0.57	3.75	0.40
Yes	4.42	0.42	3.59	0.95	4.33	0.60	3.47	0.66	3.82	0.64
*t* (*p*) value	−0.04		−0.54		−0.01		−0.18		−0.33	
*p* value	0.96		0.58		0.98		0.85		0.73	
experience of studying abroad ^a^										
No	4.39	0.42	3.41	0.62	4.31	0.46	3.41	0.57	3.74	0.40
Yes	4.60	0.43	3.50	0.65	4.45	0.43	3.54	0.67	3.88	0.49
*t* (*p*) value	−1.32		−0.34		−0.83		−0.61		−0.89	
*p* value	0.18		0.73		0.40		0.54		0.37	
Service division ^b^										
Internal medicine	4.41	0.46	3.52	0.60	4.33	0.40	3.55	0.58	3.83	0.40
Surgery	4.38	0.39	3.33	0.65	4.30	0.52	3.30	0.60	3.67	0.42
Obstetrics & Gynecology	4.52	0.50	3.54	0.64	4.37	0.48	3.54	0.43	3.85	0.36
Critical care units	4.44	0.41	3.41	0.56	4.35	0.38	3.41	0.57	3.76	0.42
F (*p*) value	0.29		0.56		0.07		1.04		0.95	
*p* value	0.83		0.64		0.97		0.37		0.42	

^a^ *t* test; ^b^ One way ANOVA.

## Data Availability

All relevant datasets in this study are described in the manuscript.
